# Impact Response of Monolithic and Laminated Polycarbonate Panels: An Experimental and Numerical Investigation

**DOI:** 10.3390/polym15244677

**Published:** 2023-12-11

**Authors:** Navid Ghavanini, Antonio Maria Caporale, Paolo Astori, Alessandro Airoldi, Paolo Panichelli

**Affiliations:** 1 Aerospace Science and Technologies Department, Politecnico di Milano, 20156 Milan, Italy; 2 Fédération Internationale de l’Automobile, 75008 Vernier, Switzerland

**Keywords:** polycarbonate, windshield perforation, impact response, finite element simulation, laminated polycarbonate

## Abstract

This study aimed to investigate the impact resistance of monolithic and laminated polycarbonate plates for windshields in motorsport applications through a coupled experimental–numerical study. Both low- and high-velocity impact tests were performed by using a drop tower and a gas gun, respectively, considering a sharp-edged projectile impacting on flat panels. The response of the polycarbonate plates was evaluated in terms of the failure mode, perforation velocity threshold, and energy absorption mechanism. The experiments allowed for the assessment and the generalization of a 3D finite element modeling approach originally developed for supersonic application based on different state-of-the-art constitutive theories, including temperature-dependent and rate-dependent von Mises plasticity coupled with ductile damage, Mie–Grüneisen equation of state, and temperature variation due to energy dissipation under adiabatic assumptions. The approach was completed with a cohesive zone model for a laminate plate and studies were performed to highlight the relevancy of different aspects of material characterization. The tests and numerical analyses performed at different velocity ranges highlight the importance of viscoplastic behavior in a polycarbonate windshield. The numerical approach showed its capability to model the different failure modes for monolithic and laminated panels and capture the perforation velocity thresholds with appreciable accuracy, which were actually found to be quite similar for the two types of panels in the test conditions considered. A numerical investigation suggests that the development of delaminations could lead to the improved energy absorption of laminated polycarbonate. To further assess the numerical model, it was used to successfully predict the penetration threshold velocity of a polycarbonate windshield subjected to a gas gun impact test.

## 1. Introduction

The usage of lightweight and durable materials is a crucial factor in the design of the protective windshields of race cars. The materials used must provide adequate safety for the drivers and must have proper impact and abrasion resistance to flying debris and objects hitting the windshields. Throughout many studies, different materials were proposed for this application, and their performances were tested. Polymers are among the materials that attracted attention due to their high durability, great strength-to-weight ratios, and excellent rate-dependent mechanical properties [1,2]. Indeed, while having low density, glassy polymers were found to have high dynamic compressive strength and toughness against impacts because of their intrinsic amorphous features [3]. In particular, polycarbonate (PC) is a resilient thermoplastic polymer that has replaced more conventional materials, such as acrylic polymers and laminated glass, by offering high impact resistance and anti-shattering behavior while also providing an excellent grade of optical transparency. A large number of works were carried out to characterize and study the mechanical behavior of PC under various loading conditions. The dependency of the PC on temperature, strain rate, and pressure was studied over a wide range of variations of these parameters and various test configurations, such as compression and tensile tests [4,5,6,7,8]. Focusing on the impact response, Wright et al. [9] experimentally studied the perforation of PC plates and classified five mechanisms observed during the penetration. Li and Goldsmith [10] addressed the impact response of thin steel and PC plates by considering blunt-shaped cylindrical projectiles. For the tests on the PC targets, failure modes in the experimental oblique impact tests were identified and discussed. In Shah and his colleagues’ work [11,12], single- and multiple-impact tests were performed on clamped circular and rectangular PC plates. In their tests, different locations on the plate were targeted, and the effect of the distance from the center on the failure and penetration was investigated. Dorogoy et al. [13] studied the dependence of the PC failure strain on triaxiality by performing impact tests on specimens confined to conical steel rings. Xu et al. [14] investigated the effect of residual thermal stresses generated during the molding process on the low-velocity impact response of PC. Furthermore, Stecconi et al. [15] addressed the issue of the reliability and statistical dispersion of the ballistic limit. In 2013, Patalak and Gideon from NASCAR^®^ (National Association for Stock Car Auto Racing, LLC (Daytona Beach, FL, USA)) demonstrated the increased impact resistance of laminated polycarbonate with respect to the monolithic one during a campaign of experimental impact testing on motorsport windshields [16].

Considering the numerical approaches for predicting the impact response of polymeric materials, a significant number of works focused on thermoplastic polymers and the development of physically based constitutive models that considered the effects of the strain rate and temperature on the deformation response. Dorogoy et al. [17] and Rosenberg et al. [3] proposed accurate numerical models to predict the ricochet and deflection of the projectile during the inclined impacts on thick polymethylmethacrylate (PMMA) plates. In Dorogoy’s study [17], it was observed that combined brittle (spalling) and ductile failure mechanisms could be employed in the numerical analyses to validate the experimental results. Moreover, they numerically studied the effect of various masses, velocities, and impact angles on the projectile’s trajectory and kinetic energy. Other models are able to capture the highly nonlinear stress–strain behavior of the polymers, including the yield-peak and post-yield softening [18]. Among these models, the Arruda Boyce 3D temperature-strain-dependent viscoplastic constitutive law, which is based on the polymers’ macromolecular structure, was found to be a valid constitutive model in representing the strain hardening and softening behavior of glassy polymers, specifically PC and PMMA, under compression and tensile loading [19]. This model is capable of predicting large inelastic deformation by taking into account the effect of the strain rate, temperature, and pressure in low-strain rates. However, as stated in reference [20], this model is not very accurate in capturing the polymer yield stress at high strain rates. In this regard, other constitutive models, such as the Drucker–Prager model, which considers the pressure dependency of the polymer, were proposed by some research studies [21], or the Johnson–Cook law, which was validated on PC panels by Sarıkaya et al. [22] and defines an experimental and numerical protocol for the identification of all the parameters of the model. Other advanced material models were developed for thermoplastics, as in Bergström and Bischoff’s work [23], where it was found that traditional metal plasticity models are not accurate for predicting the deformation of ultra-high molecular weight polyethylene (UHMWPE). Hence, they presented an advanced thermomechanical constitutive model that was found to be capable of predicting the nonlinear time- and temperature-dependent response of the material.

Focusing on PC, the failure and fracture behaviors of such material were investigated in numerous studies [24,25,26]. Estevez et al. [27] investigated the effects of viscoplasticity and crazing mechanisms on the fracture toughness of glassy polymers. A constitutive law was adopted to capture the characteristic behavior of the post-yield and the strain hardening of the polymer. Craze initiation and micro-crack propagation were modeled using the cohesive surfaces and traction-separation law. It was found that the crazing width and its opening rate are the key factors in determining the polymer fracture toughness, shear yielding, and brittle failure. In earlier primary studies [28,29], the fracture toughness values of the PC samples were obtained using a different approach via tensile and Charpy impact tests. The effect of different notch tip radii on the impact behavior of PC specimens was studied. It was found that specimens with blunt notches show a different failure mode and provide constant fracture toughness values at a higher level. Based on the findings from previous investigations, it was concluded that ductile and brittle fractures are two types of fracture mechanisms that PC exhibits, depending on the geometry and test configuration. Ductile behavior was noted when considerable plastic elongation was induced by shear yielding during the compression and tensile experiments. In contrast, it was observed that brittle fracturing could emerge in the high triaxiality state of the plain strain condition [30]. At the structural scale level, continuum finite element (FE) models were applied to represent the behavior of PC elements. In particular, phenomenological models, which were typically composed of elastic springs and viscoplastic dashpots configured in parallel networks, were used to describe the rate-dependent elastoplastic response of polymers over a wide range of strain rates [31]. These models were shown to be precise in accurately reproducing the viscous and nonlinear viscoelastic–plastic behavior of polymers. Other studies were conducted to model the fracture behavior of PC by using physically based constitutive models and failure criteria [32,33,34]. These continuum-level FE models were shown to be efficient quantitative tools to predict damage initiation and evolution to the structure’s failure and element removal point.

This literature survey indicates that a relatively large number of studies demonstrated the effectiveness of PC for protective transparent barriers and provided constitutive models for the design of effective windshields. Such models are characterized by different levels of complexity and difficulty of calibration. Attention in this study was focused on the model presented by Dorogoy et al. [35] and already applied in different cases [36], where a continuum-level FE model based on a state-of-the-art viscoplastic model integrated with a damage law and a Mie–Grüneisen equation of state was used successfully to capture the behavior of thick PC subjected to inclined impacts at a very high impact speed (about 750 m/s).

The first objective of the activities presented in this paper was to carry out tests to quantitatively assess the resistance to perforation of PC panels in a worst-case scenario for motorsport application, that is, in impacts with sharp bodies at velocities in two different ranges, by using two different experimental apparatuses. Moreover, two different engineering solutions were tested for windshield applications: a monolithic PC and a laminated PC (LPC) plate, which was obtained by gluing two PC sub-laminates with a polyurethane (PU) adhesive interlayer. A second objective consisted of the assessment of the validity of the approach followed in [35] for supersonic impacts, which was completed using a cohesive zone model (CZM) for the LPC plates, for significantly lower impact velocities by considering two different velocity ranges, namely, around 7 m/s and 110 m/s. Both the experimental and numerical activities were focused on the evaluation of the minimum velocity required for the perforation of the protective panel, which provided clear and unambiguous indications regarding the energy-absorbing capability of the barrier.

After this introduction, in the second section of the paper, the conducted experimental campaign is described, which was aimed at the evaluation of the threshold perforation velocity in tests performed by using a sharp edge projectile dropped from a drop tower for low-velocity conditions and shot using a gas gun to obtain high-velocity conditions. In both cases, the impact velocity was varied to identify as accurately as possible the perforation velocity threshold for both the monolithic and laminated PC specimens. In the third section, a numerical approach based on the finite element method and implementing Dorogoy et al.’s [35] constitutive law was used to model the experimental tests of both monolithic and laminated PC panels. The approach, which was integrated using a CZM for the LPC case, was implemented in the ABAQUS/Explicit commercial FE code, where 3D adiabatic analyses were conducted. The importance of considering the equation of state and temperature-dependent properties was evaluated by performing analyses with simplified models. Then, the numerical model was used to simulate a gas gun impact test on a polycarbonate windshield to assess its capabilities in a real application. Finally, the main results of this study are summarized and discussed in the concluding remarks.

## 2. Experimental Tests

### 2.1. Drop Weight and Gas Gun Impact Tests Layout

**Figure 1:**
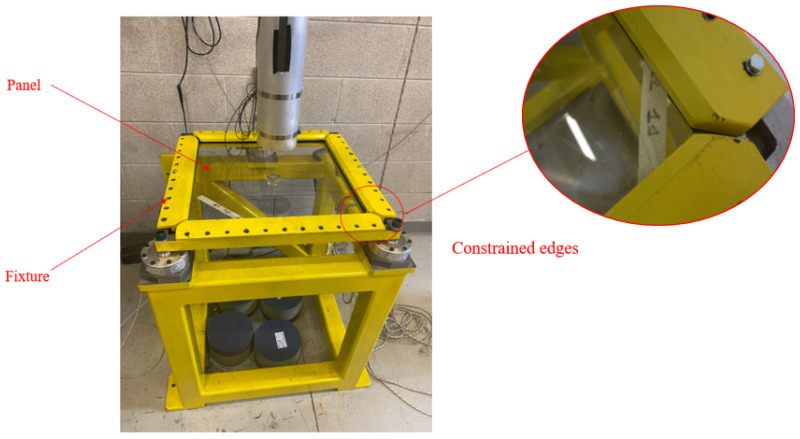
Fixture used to constrain the panels.

A campaign of experimental tests was performed to evaluate the impact response of monolithic and laminated PC flat panels in various velocity regimes. The monolithic and laminated PC specimens used were commercially available square plates with 500 mm×500 mm dimensions, with a thickness of 6.3 mm and 6.6 mm, respectively. The PC panels were made of glassy transparent Lexan PC, while the LPC ones were composed of two 3 mm thick PC plates of the same type used for the PC specimens and were bonded using a 0.6 mm PU interlayer. The impact toughness was assessed quantitively by averaging the maximum impact velocity that did not lead to the perforation of the panel and the minimum impact velocity that did. For each type of test and specimen, the damage and the shape of the indentation/perforation induced by the impactor were recorded. Due to the remarkable strain rate sensitivity of the PC, which exhibited a transient ductile-to-brittle behavior depending on the velocity of the deformation process, both the drop weight impact (DWI) and gas gun impact (GGI) tests were performed to widen the range of impact velocities and energy levels involved in the study. The target plates were clamped into a steel frame and tightened with a set of bolts to guarantee an equilibrated and repeatable solicitation of the frame on the specimens. The fixture frame, which was used for both the low-velocity DWI and high-velocity GGI tests, is shown in Figure 1.

**Figure 2:**
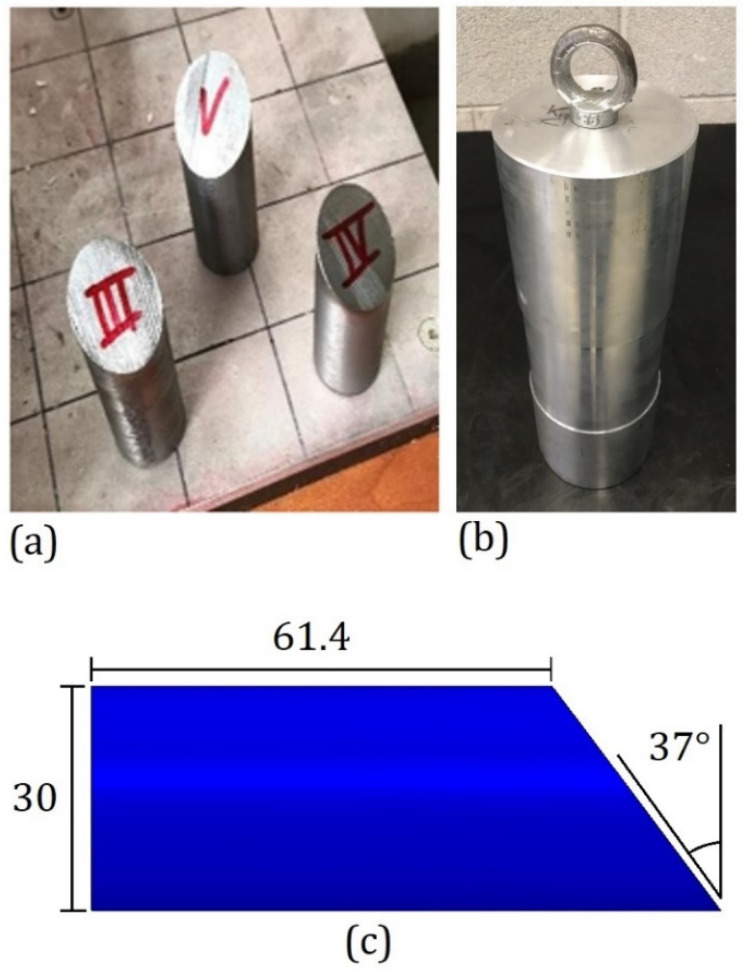
(**a**) The sharp-edge projectiles and (**b**) ballast used in the low-velocity impacts. (**c**) Profile of the projectile with measurements in millimeters.

The objective of the DWI tests was to understand the behavior of panels under low-velocity impact and find the maximum impact energy that can be absorbed without perforation for the given projectile shape and impact condition. As shown in Figure 2a, a cylindrical steel projectile with one edge slanted at 37°, a mass of 0.4 kg, and a diameter of 30 mm was used. The slanted face cut the cylinder to form a major height of 84 mm and a minor height of 61.4 mm. In the DWI tests, this sharp-edged impactor was fixed to an additional cylindrical ballast (Figure 2b) to reach a total mass of 7.66 kg. The drop tower consisted of a vertical tube driving the free fall of the projectile and ballast system, with a release system that allowed for drops from adjustable heights to obtain different impact speeds. The speed was measured by post-processing the high-speed video frames from a camera pointing at the impact area orthogonally to the impactor trajectory. During the test procedure, the impactor height varied each time. It was decreased if, in the previous test, the impactor passed through the specimen, while it was increased in the opposite case. Four PC and six LPC specimens were tested, with impact velocities in the range of 7.2–10 m/s, corresponding to kinetic energies in the range of 82.5–198.3 J. The results of the tests are reported in the subsequent Section 2.2. The high-velocity impact tests were performed by using a gas gun, which could accelerate an impactor up to a range of velocities that was one order of magnitude higher than the ones reached in the drop weight impact tests, and thus, being even more representative of debris impact against an automotive windshield. As mentioned in the introduction section, PC shows a high strain rate sensitivity, making the GGI tests fundamental for a complete characterization of the windshield material. As in the DWI tests, the objective was to identify the threshold impact velocity required to perforate the flat samples to have an unambiguous measure of the maximum energy that could be absorbed in high-velocity impact conditions.

**Figure 3:**
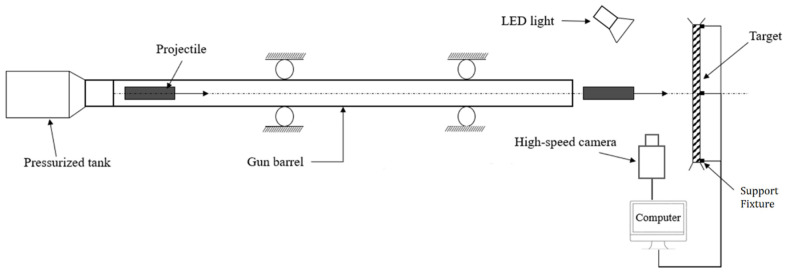
Schematic illustration of the gas gun shot.

The gas gun used in the tests consisted of a pressurized tank joined to a barrel, with a thin brass diaphragm at the junction obstructing the flow. By means of a remote-controlled trigger, a spring mechanism was released, and the brass diaphragm was perforated so that the compressed air could flow through the barrel, accelerating the impactor. In the GGI test, the projectile without any added ballast was directly inserted in a 3D-printed sabot made of polylactic acid (PLA) filament. The speed of the impactor just before the impact was measured, like in the DWI test, by post-processing the high-speed video frames from a camera pointing the test area orthogonally to the impactor trajectory. The fixture that clamped the PC specimens was the same frame used for the DWI tests. The distance between the fixture and the barrel outlet was approximately 20 cm. A functional layout including all the apparatus and systems used in the test is shown in Figure 3.

The PC and LPC specimens used for the GGI tests had the same geometry and dimensions as the ones used for the DWI tests. In the GGI tests, the tank pressure was the parameter tuned in order to adjust the impactor velocity. The tests were conducted with the same criterion as the drop tower tests: the pressure was decreased if, in the previous test, the impactor passed through the specimen and increased in the opposite case.

Ten PC specimens and five LPC specimens were tested, with velocities in the range of 32–108 m/s and, consequently, kinetic energies in the range of 210–2333 J. The results are reported in the subsequent Section 2.2.

### 2.2. Drop Weight and Gas Gun Impact Tests Results

**Figure 4:**
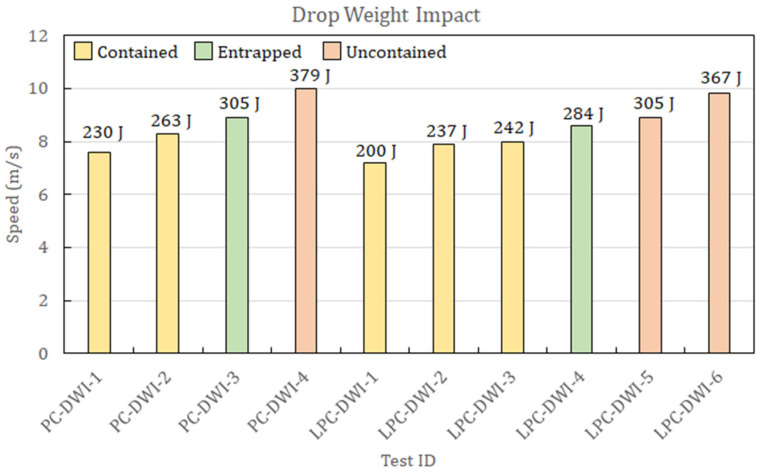
Results of drop weight impact tests on PC and LPC panels showing the outcome of every test depending on its velocity. Impact energies are reported above the column of every test.

**Figure 5:**
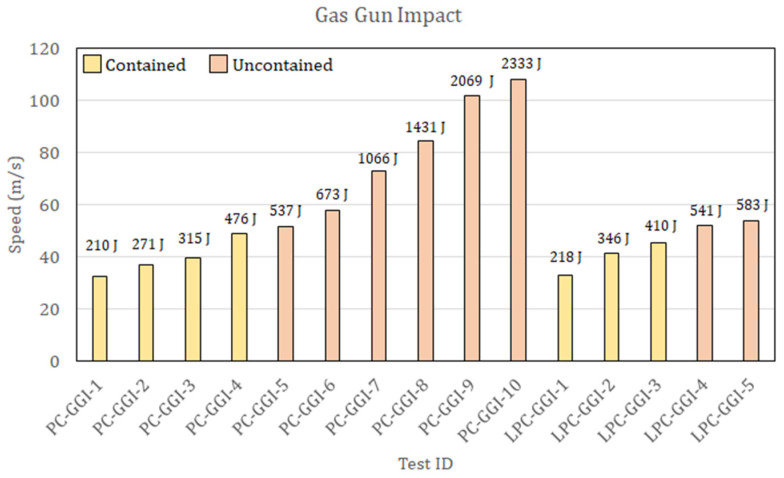
Results of gas gun impact tests on PC and LPC panels showing the outcome of every test depending on its velocity. Impact energies are reported above the column of every test.

The experimental activity performed on PC and LPC led to identifying the energy threshold between uncontained and contained impacts. During the DWI test procedure, for both materials, the presence of the ballast shown in Figure 2b did not allow the projectile to pass through in case of a complete perforation. However, outcomes with the ballast edge in contact with the panes were considered as a perforation of the plate (uncontained outcomes), while rebound cases were easily identified. Moreover, in the test campaign, the impactor became entrapped in the specimen at a specific impact velocity, with the ballast still at an apparent distance from the plate, thus allowing for precise identification of the threshold perforation velocity. During the GGI tests, such an outcome did not occur, and thus, the energy threshold was defined as the average between the energy of the highest of the contained impacts and the lowest of the uncontained ones. The results of the DWI tests and the GGI tests are summarized in Figure 4 and Figure 5, respectively, where the test outcomes are also reported.

According to the results reported in Figure 4, in DWI tests, the threshold perforation velocities and energies are reported in Table 1.

**Figure 6:**
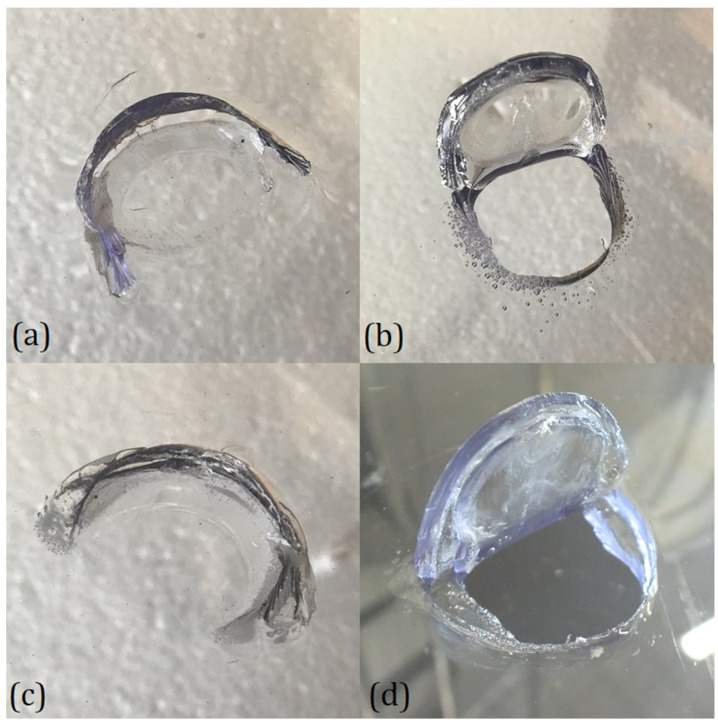
Comparison of the indentation morphologies for DWI tests: (**a**) contained PC, (**b**) uncontained PC, (**c**) contained LPC, and (**d**) uncontained LPC.

**Figure 7:**
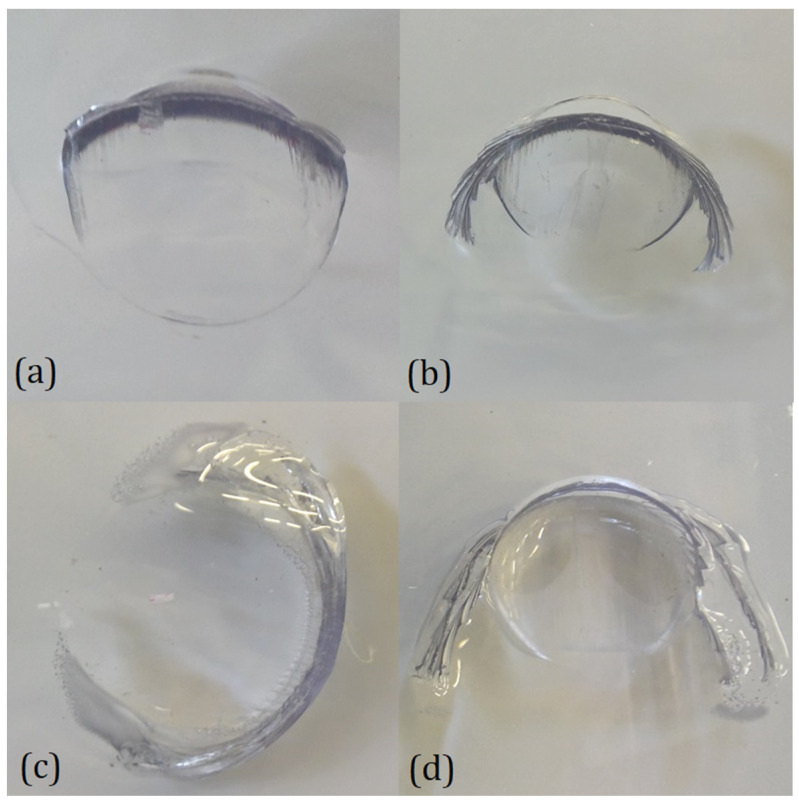
Comparison of the indentation morphologies for GGI tests: (**a**) contained PC, (**b**) uncontained PC, (**c**) contained LPC, and (**d**) uncontained LPC.

Thus, the two types of panels show very little difference in terms of energy absorption in this type of test, even if there were significant differences in the morphology of the indentation and the failure mode for the two different materials and the two different types of tests. A comparison of the different indentation morphologies is presented in Figure 6 for DWI and in Figure 7 for GGI tests.

The shape of the damage was a circular arc-shaped cut that defined a flap that was usually bent by the impactor itself. The length of these arcs depended on the type and outcome of the tests. In the PC panels, distinctive features could be observed in the indentation morphology obtained in the two tests. In fact, comparing the contained case for the two tests (Figure 6b and Figure 7b), it can be noted how the DWI test led to a bigger angle of opening of the flap than in the GGI case. This phenomenon could be explained by the fact that in uncontained DWI tests, the ballast opposed the passage of the projectile, which kept the flap open, leaving time for relaxation phenomena to occur. In all the PC cases, a distinctive series of circa-parallel fractures with a feather-like shape could be observed, starting from the extremities of the cuts, which are clearly visible in Figure 7b. This could be addressed by a series of brittle fractures that developed during the penetration. Moreover, in the DWI case (Figure 6b), some bubbles were apparent in the vicinity of the cuts, which were possibly a symptom of its thermal degradation that could have been caused by the rising temperature due to the viscous behavior of the material. In general, the LPC specimens showed less defined cuts than the PC ones, both in the DWI and GGI tests, as evident by comparing Figure 6c,d with Figure 7c,d. LPC failure tended to be characterized by delaminations developed inside the PU interlayer (Figure 6d), which were particularly pronounced at the end of the cuts. Accordingly, it can be stated that, after the initial cut, the two layers of the PC tended to separate and bend independently. Also, in this case, the DWI specimens presented a bigger angle of opening of the flap. It is worth noting that even if the damage mechanisms of the two types of panels were shown to be different, the overall energy absorption capability seemed to be at the same level for the considered test conditions. A possible explanation could rely on the fact that the higher energy dissipated by the delamination in the LPC was compensated for in the considered test conditions by the lower energy required to bend the sub-laminates separately.

## 3. Numerical Model

### 3.1. Geometries, Meshes, and Boundary Conditions

**Figure 8:**
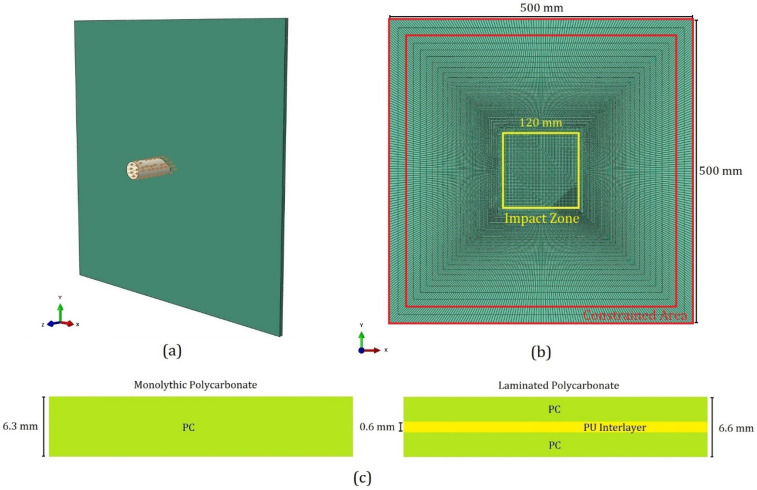
(**a**) FE assembly model of the simulation, (**b**) mesh and dimension of flat panels with impact zone (in yellow) and constrained area (in red), and (**c**) the thicknesses and schematic demonstration of the PC (left) and LPC (right) panels.

The low- and high-velocity impact simulations of a sharp-edge projectile in the center of the PC and LPC panels were carried out by representing the geometry assembly shown in Figure 8a. As in the tests, the dimensions of the modeled panels were set equal to 500 mm×500 mm (Figure 8b), and total thicknesses of 6.3 mm and 6.6 mm were adopted for the PC and LPC, respectively (Figure 8c). As is schematically illustrated in Figure 8, the PC panel was considered as a single thick layer of monolithic PC, which was modeled by using hexahedral elements, while the LPC panel consisted of two 3 mm layers of PC, which were assembled by means of a PU adhesive interlayer. The two 3 mm thick layers of LPC panels were modeled by using hexahedral elements, as in the monolithic case, and the behavior and interfacial failure of the interlayer were represented by using cohesive elements. More details about the numerical model of the LPC are given in Section 3.4. As shown in Figure 8b, a 120 mm long impact zone partition was created in the center of the target panels, and a finer mesh was seeded in this region to better capture the deformation during the impact. However, to lower the computational time of the explicit analysis, coarser elements were generated for the areas outside the impact zone by prescribing bias seeding along the panels’ diagonal lines. To find the appropriate size of the elements for the impact zone, a mesh size sensitivity analysis was carried out. Based on the convergence of the numerical results, the element size of 1.2 mm was considered for the central impact partition. Considerations of the mesh size sensitivity, which were strongly related to the material models used, are presented in Section 3.3.4. Six hexahedral elements through the thickness were considered in the simulations for the monolithic PC, whereas four elements were adopted for each of the two sub-laminates in the LPC panels that were joined by the additional layer of cohesive elements. In the final version of the mesh adopted, the PC model consisted of 99,360 8-node linear brick elements with reduced integration scheme elements (C3D8R elements [37]). In the LPC model, a total number of 149,040 elements were generated, and 16,560 single-element through-thickness layers of cohesive elements (CHO3D8 Element [37]) were interposed between the models of the two sub-layers.

**Figure 9:**
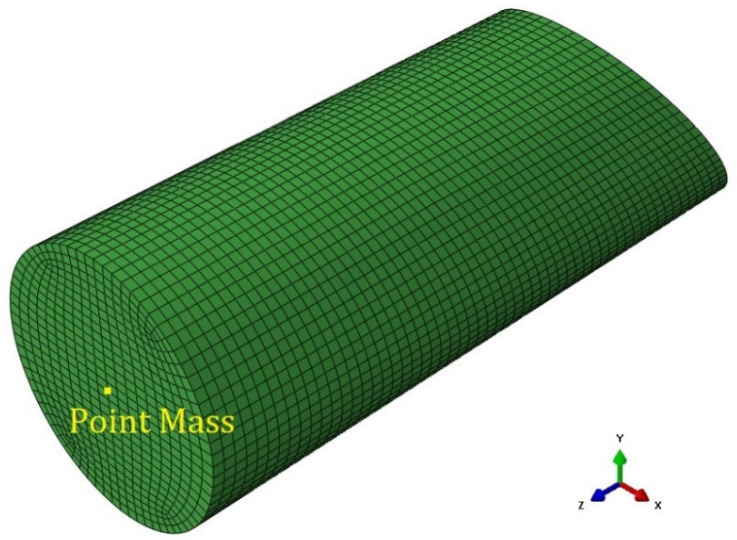
Mesh and boundary conditions of the projectile used in the FE model with the point mass used in the simulation of DWI test only.

The 400 g steel projectile was modeled according to the geometry given in Figure 9, and the dimensions previously specified. The mesh of the projectile was developed by using 11,152 hexahedral C3D8R elements. A gap of 0.001 mm between it and the plates was considered to avoid the initial contact of surfaces at the start of the analysis.

In both the drop weight and gas gun tests, the panels were constrained by inserting their edge into the slots between the clamping fixtures shown in Figure 1. Such a boundary condition was represented by including in a rigid body the elements belonging to a 40 mm frame at the border of the panel, as highlighted in Figure 8b. The degrees of freedom of these elements were restrained by applying the boundary condition to the rigid body’s reference point (RP), which was set in the middle of the target panels. The general contact algorithm based on the penalty method available in Simulia/Abaqus Explicit solver was used to model the contact behavior of the element-based surfaces during the analysis. Both the transmission of the shear and normal contact forces were considered in the interaction between the surfaces in contact. A default “hard” contact [37] was used to define the normal contact behavior; a penalty friction formulation, which was based on the Coulomb friction model, was used for the contact tangential behavior. Based on the correlation between numerical and experimental results in terms of the threshold velocity and previous studies done on the friction properties of PC and steel [38,39], friction coefficients of 0.1 were considered for both the high-velocity and low-velocity simulations. This adopted value is inside the range of values associated with PC in [39]. In the simulations, initial velocities were given to all the nodes of the projectile. For the gas gun simulations, only the impact velocity normal to the target was assigned to the impactor, which was set free to deviate from the initial trajectory during the analysis. In the drop weight impact simulations, aside from the given initial velocity, the projectile’s nodes were also confined to translate only in the normal direction to the panels throughout the whole duration of the simulation to represent the constraint of the tubular guide. Moreover, to reach the actual 7.66 kg weight used in the drop weight tests thanks to the ballast, an additional point mass element was included in the projectile model, as shown in Figure 7.

### 3.2. Adiabatic Condition

An adiabatic analysis procedure, which is available in the Simulia/Abaqus Explicit solver code, was chosen to take into account the heating effects that originated due to inelastic dissipation at the local level without modeling any heat transfer. An adiabatic analysis is usually employed in high-speed simulations where significant inelastic strain and deformations cause local heating in the material, thus leading to a variation in mechanical properties. No heat conduction was represented since it was assumed that the heat did not have the time during the impact to be transmitted inside the material. In the adiabatic calculations, the temperature rise is directly computed at each integration point of the element, converting the energy dissipated by plasticity in a heat flux per unit volume according to the following Equation (1) [37]:

(1) $$r^{pl}=\rho C_{p}\left(\theta \right)\dot{\theta }=\varphi \sigma :{\dot{\varepsilon }}^{pl}$$

where $r^{pl}$ is the heat flux, ρ is the mass density, *C_p_* is the specific heat, *θ* is the temperature, φ is the constant user-defined inelastic heat fraction, σ is the stress, and ${\dot{\varepsilon }}^{pl}$ is the plastic strain rate. The temperature rise calculated using Equation (1) is used to vary the property of the PC according to data provided in the characterization.

### 3.3. PC Constitutive Laws

The characterization tests described in the literature show that PC behaves as a ductile material, even at high strain rates, i.e., 1200 s^−1^ to 8000 s^−1^ [7,8,35,40,41]. The typical response of PC is characterized by an initial elastic response followed by yielding, an initial strain softening, and then by a plastic strain hardening phase until a final failure. In the present study, the Lexan-type PC, which was used for the tested panel in the experimental phase, was modeled by using the constitutive law proposed by Dorogoy et al. [35]. This constitutive law was found to be very effective at modeling impacts on thick PC plates with velocities around ~750m/s. The corresponding details of the adopted constitutive law are summarized in Table 2. The physical and elastic properties of PC and steel used in the model are reported in Table 3.

#### 3.3.1. Non-Linear Behavior

Because of this evidence, the constitutive law includes an elastic–plastic strain-rate and temperature-dependent von Mises plasticity model and a Mie–Grüneisen equation of state to represent the deviatoric and volumetric constitutive behaviors of the PC, respectively. The plasticity data implemented in the Abaqus Explicit solver were defined by direct entry of tabular tensile test data at different temperatures and strain rates in terms of the yielding stress on plastic equivalent strain curves. The yield stress at a given strain, strain rate, and temperature is interpolated directly from these data [37]. The Mie–Grüneisen relates the pressure p in a solid with the volumetric strain η. The development of this law requires the introduction of Hugoniot equations for shock-compressed fluid, which linearly links the velocity of the shock wave $U_{s}$ and the velocity of particles $U_{s}$ in the following form:

(2) $$U_{s}=c_{0}+sU_{p}$$

where $c_{0}$ is the bulk sound speed and s is the slope in $U_{s}$ versus $U_{p}$ diagram. The parameters defined above, together with $E_{m}$ the internal energy per unit mass, allowing for expressing the final form of the Mie–Grüneisen equation, which reads

(3) $$p=\frac{\rho_{0}c_{0}^{2}\eta }{{\left(1-s\eta \right)}^{2}}(1-\frac{\Gamma_{0}\eta }{2})+\Gamma_{0}\rho_{0}E_{m}$$

#### 3.3.2. Damage Models

Modeling of the perforation of the projectile in the PC and LPC was obtained by progressively damaging and finally eroding elements beyond certain damage thresholds. Two types of failure models, namely, ductile damage criteria and tensile failure, were considered. Ductile failure was associated with the plastic strain developed in the deviatoric response of the material, which was modeled using conventional von Mises plasticity. A scalar damage variable was used to degrade the stress predicted by the elastic–plastic model, with a given threshold and evolution law. Tensile failure was adopted to eliminate the elements once a hydrostatic tensile stress value was exceeded. This approach allowed for representing both the variation of the failure strain with strain rate for the onset of ductile failures, as well as the brittle behavior of the material at high strain rates. Considering that in the present case, the geometry and boundary conditions differ significantly from the one where this PC constitutive law was applied, the need to include the equation of state and adiabatic conditions with temperature-dependent properties was assessed by simulating the impacts without such features. Accordingly, two simplified models were evaluated: the first used a PC law that excluded the Mie–Grüneisen equation of state, whereas the second excluded the adiabatic condition, and thus, the temperature variation of the material properties. The three models were named EOS, No-EOS, and No-ADB, respectively. Moreover, a sensitivity study on the values adopted for the friction coefficient was included.

#### 3.3.3. Model Parameters and Calibration

The material data used were taken from [35]. The density and elastic constants of the PC and steel material of the impactor are presented in Table 3. The table also reports the specific heat and inelastic heat fraction φ used in the simulation. The material of the projectile was considered purely elastic since no plastic deformation or damage was detected in the tests.

**Table 3:** Physical and elastic properties used in the modeling (taken from [35]).

Material	*ρ* [kg/m^3^]	ν	G [MPa]	*C_p_* [J/kg·K°]	Inelastic Heat Friction *φ*
PC	1200	0.3	803 at T = 0° 80.3 at T = 200°	1300	0.9
Steel	7800	0.37	76,600	420	1.0

**Figure 10:**
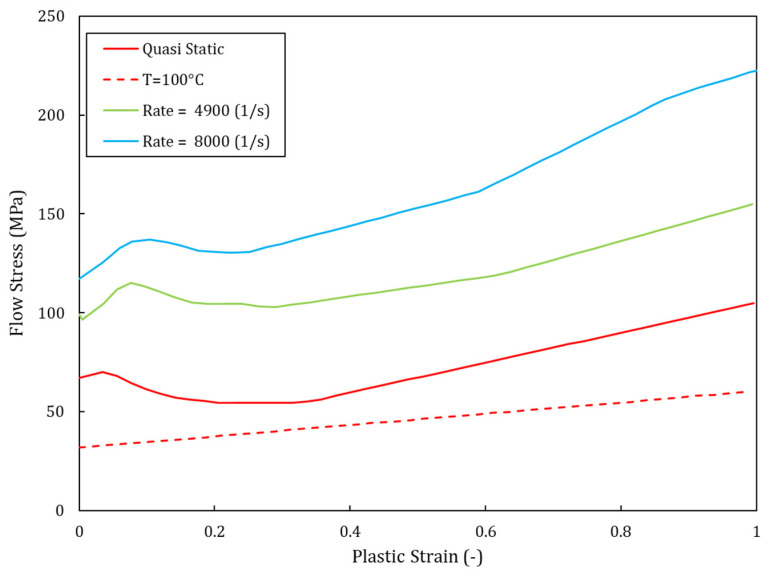
True stress–true strain and plastic properties of the PC used in the numerical analyses (from data presented in [35]).

The damage and failure values used in the simulations are presented in Table 4, which includes the yielding stresses, the damage initiation data, and the fracture strain and strain rate. The value of the failure displacement ${\bar{u}}_{f}^{pl}$ reported is a mesh-dependent parameter, which was set to the value suggested in [35], considering that the size of the elements adopted in that case was similar to the one used in the present study. Further consideration of this parameter is presented in Section 3.3.4. The data used to calibrate the strain-rate- and temperature-dependent hardening curves of the von Mises plasticity model are shown in Figure 10, while the Mie–Grüneisen data are presented in Table 5. It is worth noting that the curves presented refer to Split–Hopkinson bar tests with material undergoing compressive stress states, whereas the strain at the damage onset reported in Table 4 was selected from [35] to represent ductile failures in tensile stress conditions. It can also be observed that the hydrostatic cut-off stress of 150 MPa was set at a very high stress level, close to the maximum von Mises stress levels experienced by the material at a strain rate of 8000 s^−1^. Accordingly, the cut-off was not expected to be activated unless severe stress triaxiality or very high strain rates were reached in the material.

**Table 4:** Failure and damage values used for the PC (taken from [35]).

Yielding Stress			Ductile Failure			Tensile Failure
Strain Rate $\dot{\varepsilon }$	*T*	$\sigma_{y}(T,\dot{\varepsilon })$	Damage Initiation		Damage Evolution	Hydrostatic Cutoff Stress $\sigma_{\mathrm{cutoff}}$
			Strain Rate $\dot{\varepsilon }$	Fracture Strain $\varepsilon^{pl}$	Failure Displacement ${\bar{u}}_{f}^{pl}$	
1/s	°C	MPa	1/s	-	μm	MPa
0.001	25	67.3	0.001	1	80	150
4900	25	99.3	4900	0.85		
8000	25	117.2	8000	0.85		
0.001	125	32.4	80,000	0.6		

#### 3.3.4. Mesh Size Dependence

The convergence of numerical results by decreasing the mesh size is guaranteed as a consequence of the ellipticity of the finite element differential problem [42]. Among the possible reasons for the loss of ellipticity is the strain-softening behavior of materials, which leads to severe mesh-size-dependent strain localization and a lower volumetric fracture energy for smaller elements [37]. To mitigate this effect, the damage laws included have to be normalized on the element’s characteristic length.

**Figure 11:**
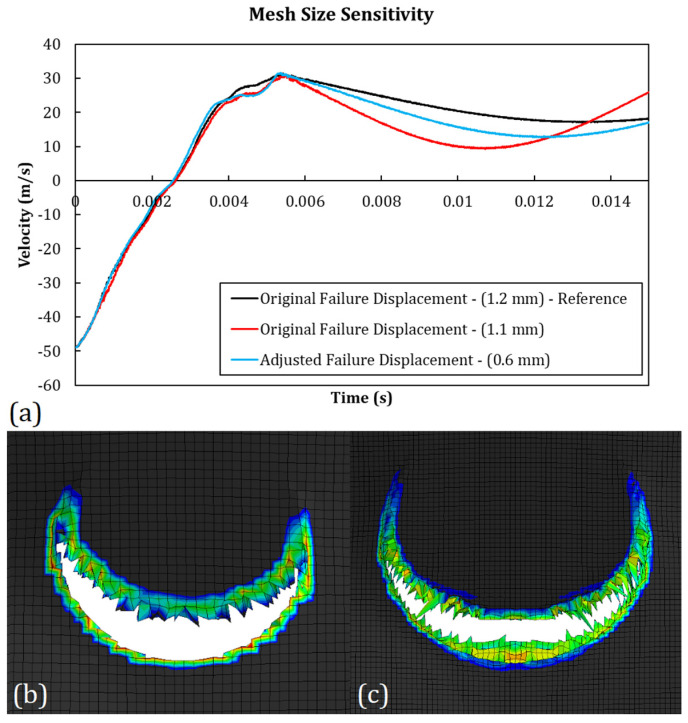
(**a**) Results of the mesh size sensitivity study: (**a**) numerical projectile speeds for PC-GGI-4 test with different meshes and (**b**,**c**) comparison between spatial gradients of plastic equivalent strain, both at time $t=1.5\times 10^{-3}\;s$ but with different meshes.

It is worth noting that in the present case, both the damage and plasticity models (see Figure 10) introduced strain softening in the material. Although the damage law can be adjusted to mitigate the mesh size effects by varying the failure displacement ${\bar{u}}_{f}^{pl}$, the localization effect due to the strain softening regime included in the plasticity hardening law remains. The results of a sensitivity study performed using the PC-GGI-4 case are reported in Figure 11. The original curve, which was obtained with elements with a squared area with a side of 1.2 mm, was compared with the one obtained with elements with a side of 1.1 mm and 0.6 mm. The first one was obtained by keeping the original value of failure displacement (80 μm), while in the second case, the failure displacement had to be tuned to obtain a good result. The optimal value of the failure displacement found was 830 μm. In the first phase of the impact, when the projectile was indenting the panel, the curves showed an almost identical behavior. Small variations were observed in the second phase when the projectile rebounded and even a very small difference in its rigid rotations could lead to differences in the velocity curve. These results showed that small differences in the elements area did not lead to significant variation in the analysis results. Meanwhile, for bigger element size reductions, the failure displacement had to be increased to avoid a loss of toughness of the elements.

In Figure 11b,c, the difference in strain localization of two analyses with different mesh sizes was reported. The contours show the spatial gradient of the equivalent plastic strain, both at time $t=1.5\times 10^{-3}\;s$, which were obtained in the case of two analyses performed on the PC-GGI-4 case with element sides of 1.2 mm and 0.6 mm, respectively. It can be seen that the adjustment in the failure displacement led to very similar contours of strain localization, though some differences were still visible.

### 3.4. Polyurethane Adhesive Constitutive Law

**Figure 12:**
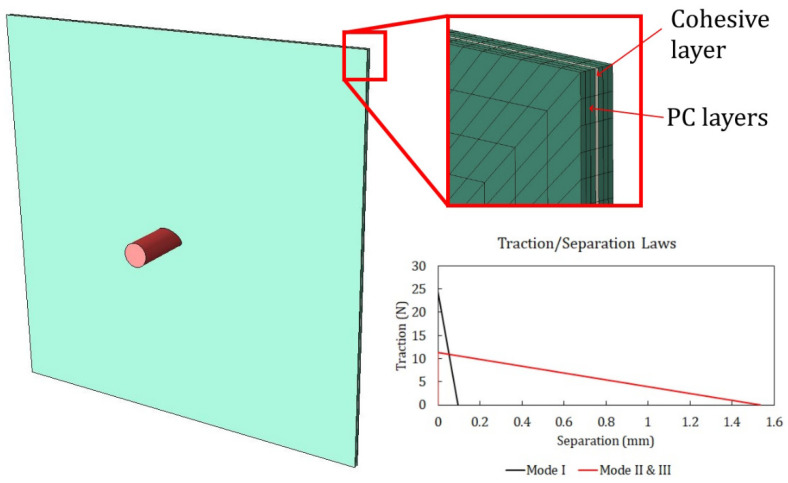
Implementation of a cohesive zone model in the FE model of laminated PC with a mixed-mode bilinear damage law.

In the impact tests on the LPC panels, the debonding of PC layers is one of the failure features that was observed during penetration. For this reason, a cohesive zone model was adopted to model the cohesive behavior of the PU interlayer at the interfaces (Figure 12). The implementation in the finite element model was carried out by adopting conventional cohesive elements available in the Simulia/Abaqus code (COH3D8 elements [37]) with a bilinear damage law.

The constitutive response of a cohesive element is represented by a relationship between the traction transmitted and the discontinuities at the interface, as sketched in Figure 12. Such a cohesive zone model (CZM) is able to model three types of interface openings, one in the normal direction of the interface layers (*n*), which is determined by normal stress, and two along the interface plane, which are driven by the shear stress in the orthogonal direction (s) and (n) and are considered characterized by identical properties. Each mode has its own strengths and toughness, which were set equal to the critical strain energy release rates $G_{n}^{c}$, $G_{s}^{c}$, and $G_{t}^{c}$. The Benzeggagh–Kenane (BK) fracture criterion is specified to model the fracture and crack propagation of delamination under mixed-mode conditions. The criterion assumes $G_{s}^{c}=G_{t}^{c}$ and define the total toughness in a generic mixed mode as in Equation (4) [35]:

(4) $$G_{n}^{c}+\left(G_{s}^{c}-G_{n}^{c}\right){\left\{\frac{G_{S}}{G_{T}}\right\}}^{\eta }=G^{C}\;\;where\;G_{S}=G_{s}+G_{t},\;G_{T}=G_{n}+G_{S}.$$

An element deletion option was included in the simulations by imposing the elimination of the cohesive elements when the damage parameter *D* reached a value of 0.999.

The properties of the CZM for the PU adhesive used in the simulations presented are given in Table 6. The toughness values were extracted from the reference [43], which obtained the cohesive properties of the PU adhesives by performing the standard characterization tests. The values of the elastic modulus *E*, shear modulus *G*, and strengths were slightly tuned, starting from the values given in [43], to better fit the morphology of the indentation. The value of the BK power η was extracted from reference [44].

## 4. Numerical Results and Discussion

### 4.1. Threshold Velocities Correlation

**Figure 13:**
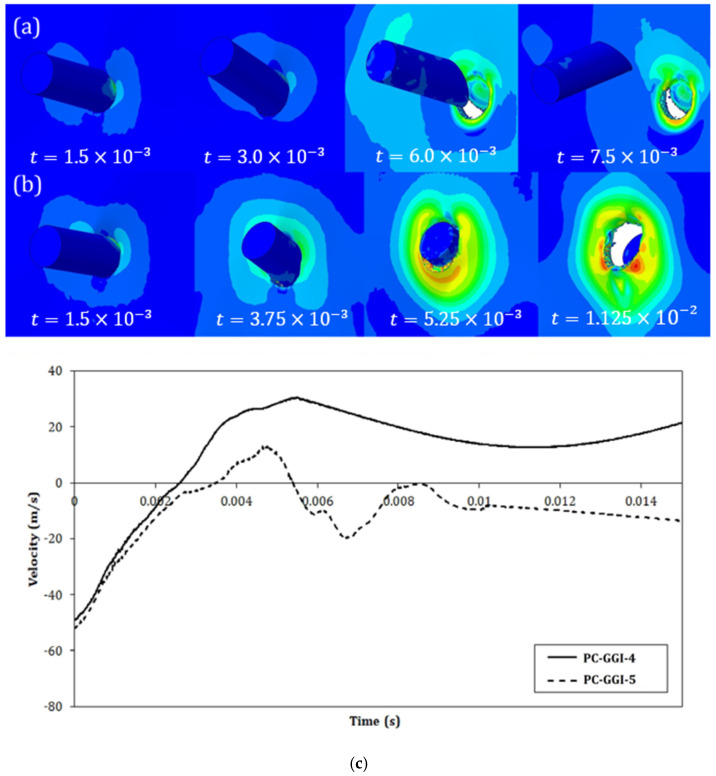
Comparison between the contours of von Mises stress in the PC panels in the numerical simulations of GGI tests: (**a**) PC-GGI-4, (**b**) PC-GGI-5, and (**c**) curves of the projectile velocity of the two cases.

The outcomes of the numerical simulations were evaluated by measuring the projectile residual velocity in the direction normal to the panel, as measured at the point shown in Figure 7. If the sign of the final velocity was concordant with the initial velocity, an uncontained outcome was obtained, while discordance in sign represented a case where the projectiles were contained, and a null velocity was referred to as an entrapped case. Examples of contours of an uncontained and contained case with the resultant velocity curves are shown in Figure 13. The results obtained with the numerical model are reported and compared with the experimental ones in Table 7. The values of the threshold velocity of both experimental and numerical tests are reported with the percentage error, which was computed between the experimental and numerical velocities of the entrapped tests, where available. As stated in the experimental Section 2, where entrapped tests were not available, the arithmetic average was computed between the highest velocity of the contained cases and the lowest velocity of the uncontained outcomes, both in the experimental and numerical cases. In those cases, the resultant threshold velocity is reported together with the uncertainty.

**Figure 14:**
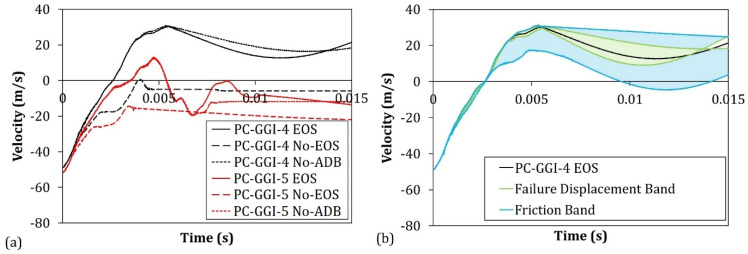
(**a**) Projectile velocity comparison between EOS, No-EOS, and No-ADB numerical models of PC-GGI-4 and PC-GGI-5 tests and (**b**) error band results of sensitivity studies on friction coefficient and failure displacement in the PC-GGI-4-EOS test.

The results reported in Table 7 show that the EOS model was capable of capturing the threshold velocities of both the monolithic and laminated PC in different velocity ranges with an error that did not exceed 7%. On the other hand, the results showed how by removing the Mie–Grüneisen equation of state (No-EOS), the error increased up to about 16%. It must be underlined that in the simulation when implementing the EOS model, the tensile failure was never triggered since no element ever reached the hydrostatic cutoff stress. Thus, the increased error in the No-EOS results could be explained by the difference in the volumetric response between the Mie–Grüneisen equation and the standard elastic material. Removing the adiabatic conditions, the obtained results did not show a significant difference in terms of the velocity thresholds. The difference in terms of the velocity curves is shown in Figure 14a, where the numerical curves of the same cases shown in Figure 13 are reported for the EOS, No-EOS, and No-ADB models. The curves obtained using the No-ADB model showed small differences with respect to the EOS one only in the final part when the bullet rebounded in the contained case or when the bullet slid through the panel in the uncontained case. On the other hand, No-EOS systematically underestimated the resistance of the material. In Figure 14b, the two bands in blue and green represent the variation in projectile velocity as a consequence of a variation of ±50% in the friction coefficient and ±12% in the failure displacement, respectively. These results showed that in the given ranges of variation, the two parameters did not influence the outcome of the test, influencing only the latest part of the curve where the lateral surface of the projectile was in contact with the panel.

### 4.2. Numerical Perforation Morphologies Observation

**Figure 15:**
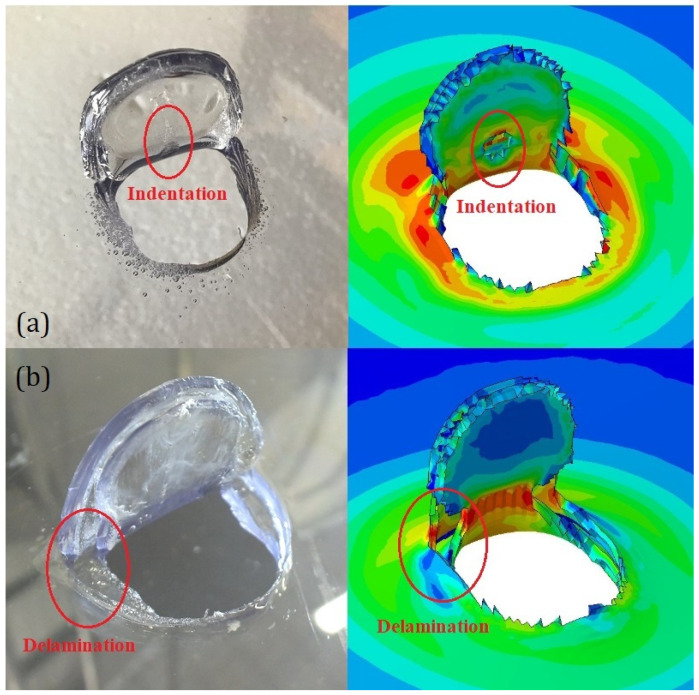
Comparison between experimental and numerical indentation morphologies for (**a**) an uncontained DWI test on PC and (**b**) an uncontained DWI test on LPC. The numerical contour show the Von Mises stress.

The numerical model was also capable of reproducing with a certain accuracy the morphologies of the different indentations. Some relevant similarities were found when comparing the experimental and numerical indentations. In Figure 15, two uncontained DWI test results on PC and LPC are presented, both of which were obtained using the EOS model. In Figure 15a, it can be noted how the model captured the small indentation on the flap due to the impact of the slanted surface of the projectile while the flap itself was already open. In Figure 15b, the capability of the model to capture the delaminations in the PU adhesive interlayer can be noted.

**Figure 16:**
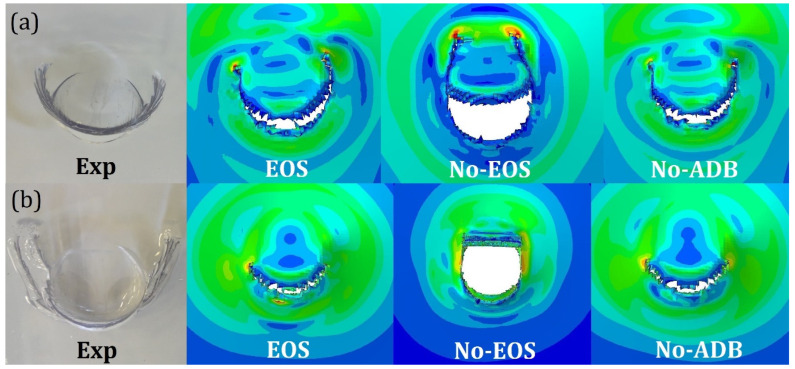
Experimental/numerical comparison between the indentation morphology in the case of an uncontained GGI test on (**a**) monolithic PC and (**b**) laminated PC for the different cases of EOS, No-EOS, and No-ADB models. The numerical contour show the Von Mises stress.

In Figure 16, the uncontained GGI tests using PC and LPC, which were obtained using the EOS, No-EOS, and No-ADB models, are presented and compared with the experimental case. EOS and No-ADB showed almost identical morphologies, further indicating that in this kind of application, the adiabatic condition was unnecessary. On the other hand, the No-EOS morphologies differed from the EOS ones, with a larger cut and opening of the flap. As an example, Figure 16a shows how in the No-EOS morphology, the cut propagated during the penetration, which was a symptom of an excessive ductile response of the material. The comparison between the EOS, No-ADB, and the experimental morphologies showed an acceptable correlation.

### 4.3. Numerical Investigation on the Difference between PC and LPC Responses

**Figure 17:**
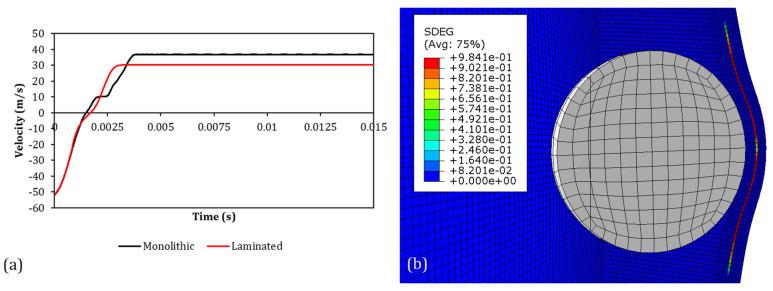
(**a**) Velocity time history of the spherical projectile impacting a PC and an LPC panel. (**b**) Transversal cut of the LPC panel being impacted. The contour shows the damage to the cohesive layer.

Both the experimental and the validated numerical results obtained in the activities described in this work indicate that monolithic and laminated polycarbonate panels may exhibit different failure modes, though the penetration threshold velocity was quite similar in both types of test conditions considered. However, the results obtained by Patalack and Gideon at NASCAR^®^ [16] showed an improved impact resistance of LPC. These differences can be explained by considering the different test conditions, which were based on a cylindrical projectile impacted on a curved windshield in [16]. Actually, the improved impact resistance of the LPC could be related to the energy absorbed in the damage mechanisms of the PU interlayer. However, the results obtained in this work seem to indicate that the debonding of the PU interlayer could have reduced the energy necessary to bend the panels, thus compensating for the possible benefit of the delamination. It is reasonable to state that the energy balance could be different in different test conditions. To verify this, numerical analyses were performed, which were identical to the ones previously described with the EOS material law but with a spherical projectile instead of the sharp-edged one, both on PC and LPC panels. The spherical projectile had the same mass and material properties as the sharp-edged one, with a resulting diameter of 46 mm. A velocity of 51.8 m/s, the same as in the PC-GGI-5 test, was imposed on the sphere. In Figure 17a, the velocity time history of the projectile is reported for the two cases.

The results in Figure 17a show that the spherical projectile was contained in both cases, but the after-impact velocity in the LPC case was 18% lower than the one in the PC case, thus showing the improved energy absorption of LPC panels. In Figure 17b, the delamination observed during the impact is also shown. If delaminations are assumed as the reason for the increased energy absorption on LPC, then it is reasonable to suppose that in an impact with a sharped-edged projectile, which tends to cut and bend the panel with a relatively small, delaminated area, this effect is less efficient, thus leading to reduced energy absorption of LPC. This could explain why the threshold velocities obtained in the present work on the PC and PCL panels showed negligible differences.

## 5. Assessment of the Model with a Windshield Test

**Figure 18:**
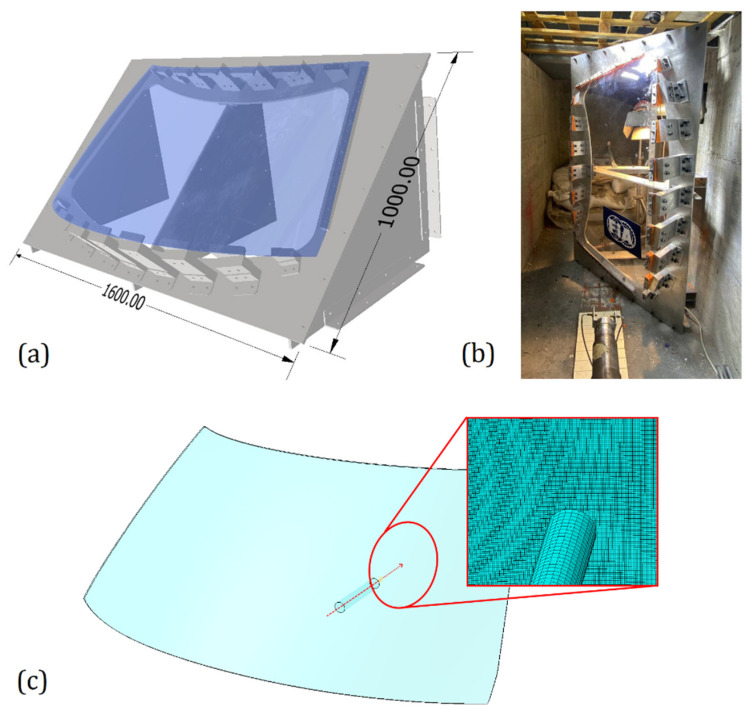
(**a**) CAD drawings of the aluminum frame used to fix the windshield, (**b**) arrangement of the windshield during the GGI test, and (**c**) geometry of the windshield and projectile used in the numerical simulations with the detail of the mesh.

The numerical model described was further validated by reproducing an experimental test on an entire windshield. The experimental test was performed on a 3 mm thick windshield made of monolithic PC. A stiff aluminum frame was designed and manufactured ad hoc to support the windshield with an angle of 45° from the horizontal plane (see Figure 18a), the angle at which it is mounted in a real car frame; forty-five holes were drilled along the edge of the windshield and corresponding M8 bolts were used to fix it on the frame, inserting aluminum plates under the bolt heads, with a thin soft pad between the plates and the polycarbonate surface to better smear the local pressure; a relatively uniform constraint was then obtained, approximating the real car installation. The projectile used had a cylindrical shape with a diameter of 30 mm, a length of 183.3 mm, and a mass of 1 kg. The projectile was accelerated using the same gas gun described in Figure 3 and shot toward the position of the driver’s head (see Figure 18b,c). High-speed cameras were positioned to measure the impact speed.

In the numerical model, the windshield was modeled with 268,410 C3D8R elements. The mesh was biased to have elements with a squared area with a 1 mm side and thickness of 0.6 mm in the impact area, but larger elements elsewhere to reduce the computational cost. The nodes of the external edge were constrained as a rigid body and all their degrees of freedom were set to zero. The steel properties described in Table 3 were used for the projectile. The previously described EOS material model was used for the windshield as well. Considering the lower thickness of the elements used, the failure displacement parameter was increased, assuming a linear relationship with the thickness, which led to the value of 140 μm. Two experimental and numerical projectile velocities, as well as the test outcome, are reported in Table 8.

**Figure 19:**
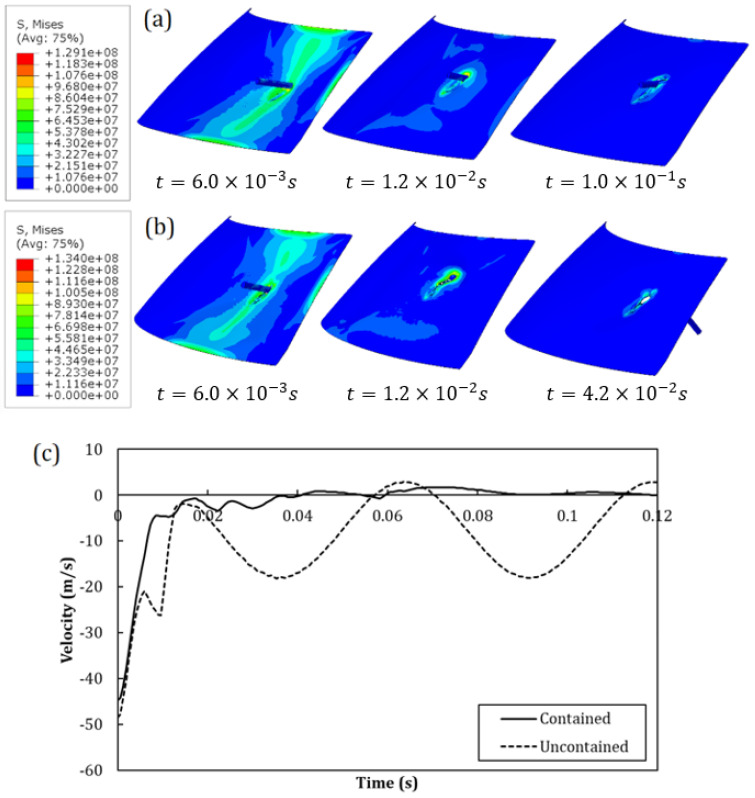
Comparison between the contours of von Mises stress in the PC windshield in the numerical simulations of GGI tests: (**a**) WS-GGI-1 (entrapped) and (**b**) WS-GGI-2 (uncontained). (**c**) Curves of the projectile velocity of the two cases.

In Figure 19a,b, the comparison between the evolution in time of two contour plots of the von Mises stress for the WS-GGI-1 test (entrapped) and WS-GGI-2 test (uncontained) is reported. In Figure 19a, the comparison in terms of the time evolution of projectile velocity is reported. Also, in this case, the proposed numerical model seemed to be able to capture the threshold penetration velocity with a certain accuracy.

## 6. Concluding Remarks

In this work, the impact response of monolithic and laminated PC subjected to a sharp projectile was studied experimentally and numerically in low- and high-velocity regimes. Monolithic and laminated panels showed similar impact behaviors from the quantitative point of view: the energy absorption and velocity needed to fully penetrate the panels were similar, both in the low- and high-velocity regimes. In contrast, from the qualitative point of view, the PC and LPC panels showed different failure mechanisms: the PC seemed to fail mostly due to ductile damage with signs of brittle failure only at the extremities of the cut, while the LPC failed for a coupled action of ductile damage and delamination of the two polycarbonate layers due to the degradation of the polyurethane adhesive. A FE model was developed inside the Abaqus/Explicit environment to reproduce the experimental tests. This model implemented a state-of-the-art constitutive law, available in the literature for PC while using a CZM for the PU adhesive. This law includes a strain-rate- and temperature-dependent von Mises plasticity coupled with ductile damage and the Mie–Grüneisen equation of state coupled with tensile failure. This model was validated in a range of energy that was considerably lower than the ones used in the original application [35]. Moreover, the relevance of temperature-dependent phenomena was evaluated by setting an adiabatic condition in the analysis. In parallel, the capabilities of a simplified model that excluded the Mie–Grüneisen equation were investigated. The numerical results showed that the numerical model with the complete constitutive law and the adiabatic condition could successfully capture with a small error the threshold penetration velocity for both PC and BCL in both velocity regimes while reproducing the indentation morphology with good approximation. The adiabatic conditions were shown to be unnecessary, both in capturing the threshold velocity and the indentation morphology, while the simplified constitutive law was shown to be inadequate in modeling the experimental tests, underestimating the resistance of the panels. A numerical investigation showed that the LPC could absorb more energy than the monolithic PC if the projectile was rounded enough, thus involving a bigger area and allowing for the development of larger delaminations. The numerical model was further assessed by simulating an experimental GGI test on an entire polycarbonate windshield. Also, in this case, the numerical model was able to capture the penetration threshold velocity with acceptable accuracy.

## Figures and Tables

**Table 1:** Threshold perforation velocities and energies.

	DWI Threshold		GGI Threshold	
PC	8.9 m/s	303 J	50.8 m/s	537 J
LPC	8.6 m/s	283 J	52.0 m/s	541 J

**Table 2:** Summary of the material laws used in the polycarbonate constitutive model.

	Non-Linear Behavior		Damage Model	
	Law	Data	Law	Data
Deviatoric behavior	Von Mises plasticity	Table 4, Figure 8	Ductile damage	Table 4
Volumetric behavior	Mie–Grüneisen	Table 5	Tensile failure	Table 4

**Table 5:** Mie–Grüneisen equation of state data for PC (taken from [35]).

*c*_0_ (m/s)	s	$\Gamma_{0}$
1933	2.65	0.61

**Table 6:** CZM parameters for the PU adhesive layer.

Property	Value
Elastic modulus $E\;\left(GPa/mm\right)$	1.79
Shear modulus G (GPa/mm)	0.25
Normal strength $t_{n}$ (MPa)	24.4
Shear strength $t_{s}\;$(MPa)	11.4
Fracture toughness in tension $G_{Ic}$ (N/mm)	1.18
Fracture toughness in shear $G_{IIc}$ (N/mm)	8.72
Benzeggagh–Kenane power *η*	1.8

**Table 7:** Comparison between the threshold velocities obtained in the experimental tests and with EOS, No-EOS, and No-ADB models.

Test	Panel Type	Threshold Velocity (m/s)			
		Experimental	Numerical (% Difference)		
			EOS	No-EOS	No-ADB
DWI	PC	8.9	8.3 (6.7%)	7.5 (15.7%)	8.3 (6.7%)
	LPC	8.6	8.0 (6.9%)	7.5 (12.8%)	8.0 (6.9%)
GGI	PC	50.3±1.5	50.3±1.5	46.9±1.9	50.3±1.5
	LPC	48.6±3.1	48.6±3.1	45.0±0.2	48.6±3.1

**Table 8:** Comparison between the experimental and numerical penetration threshold velocities obtained in the GGI tests.

Test	Velocity	Outcome	
		Experimental	Numerical
WS-GGI-1	44.8 m/s	Contained	Entrapped
WS-GGI-2	48.5 m/s	Uncontained	Uncontained

## Data Availability

The data will be available on request.

## References

[1] Sarva S., Mulliken A.D., Boyce M.C., Hsieh A.J. (2006). Mechanics of transparent polymeric material assemblies under projectile impact: Simulation and experiments. Transformational Science and Technology for the Current and Future Force.

[2] Bardia P., Narasimhan R. (2006). Characterization of pressure-sensitive yielding in polymers. Strain.

[3] Rosenberg Z., Surujon Z., Yeshurun Y., Ashuach Y., Dekel E. (2005). Ricochet of 0.3″ AP projectile from inclined polymeric plates. Int. J. Impact Eng..

[4] Mulliken A.D., Boyce M.C. (2006). Mechanics of the rate-dependent elastic-plastic deformation of glassy polymers from low to high strain rates. Int. J. Solids Struct..

[5] Bauwens-Crowet C., Bauwens J.-C., Homès G. (1972). The temperature dependence of yield of PC in uniaxial compression and tensile tests. J. Mater. Sci..

[6] Hasan O.A., Boyce M.C. (1995). A constitutive model for the nonlinear viscoelastic viscoplastic behavior of glassy polymers. Polym. Eng. Sci..

[7] Song P., Trivedi A., Siviour C.R. (2023). Tensile testing of polymers: Integration of digital image correlation, infrared thermography and finite element modelling. J. Mech. Phys. Solids.

[8] Chen L., Zhang M., Li D., Li Y. (2023). Infrared thermographic evaluation of thermal release phenomena in polycarbonate during plastic deformation. Adv. Ind. Eng. Polym. Res..

[9] Wright S.C., Fleck N.A., Stronge W.J. (1993). Ballistic impact of PC-An experimental investigation. Int. J. Impact Eng..

[10] Li K., Goldsmith W. (1997). Perforation of steel and PC plates by tumbling projectiles. Int. J. Solids Struct..

[11] Shah Q.H., Abakr Y.A. (2008). Effect of distance from the support on the penetration mechanism of clamped circular PC armor plates. Int. J. Impact Eng..

[12] Shah Q.H. (2009). Impact resistance of a rectangular PC armor plate subjected to single and multiple impacts. Int. J. Impact Eng..

[13] Dorogoy A., Rittel D. (2015). Effect of confinement of thick polycarbonate plates impacted by long an AP projectiles. Int. J. Impact Eng..

[14] Xu Y., Lu H., Gao T., Zhang W. (2015). Predicting the low-velocity impact behavior of polycarbonate: Influence of thermal history during injection molding. Int. J. Impact Eng..

[15] Stecconi A., Landi L. (2020). Finite element analysis for impact tests on polycarbonate safety guards: Comparison with experimental data and statistical dispersion of ballistic limit. ASCE-ASME J. Risk Uncertain. Eng. Syst. Part B Mech. Eng..

[16] Patalak J., Gideon T. (2013). Ballistic Testing of Motorsport Windshields. SAE Int. Transp. Saf..

[17] Dorogoy A., Rittel D., Brill A. (2010). A study of inclined impact in polymethylmethacrylate plates. Int. J. Impact Eng..

[18] Anand L., Gurtin M.E. (2003). A theory of amorphous solids undergoing large deformations, with application to polymeric glasses. Int. J. Solids Struct..

[19] Boyce M.C., Parks D.M., Argon A.S. (1988). Large inelastic deformation of glassy polymers. part I: Rate dependent constitutive model. Mech. Mater..

[20] Du S., Mullins M., Hamdi M., Sue H.J. (2020). Quantitative modeling of scratch behavior of amorphous polymers at elevated temperatures. Polymer.

[21] Esfahlani S.S. (2021). Ballistic performance of polycarbonate and polymethylmethacrylate under normal and inclined dynamic impacts. Heliyon.

[22] Sarıkaya M., Güden M., Kambur Ç., Özbek S.Ç., Taşdemirci A. (2023). Developmente of the Johnson-Cook flow stress and damage parameters for the impact response of polycarbonate: Experimental and numerical approach. Int. J. Impact Eng..

[23] Bergström J.S., Bischoff J.E. (2010). An Advanced Thermomechanical Constitutive Model for UHMWPE. Int. J. Struct. Chang. Solids.

[24] Ishikawa M., Noarisawa I., Ogawa H. (1977). Criterion for Craze Nucleation in PC. J. Polym. Sci. Polym. Phys. Ed..

[25] Nimmer R.P., Woods J.T. (1992). An investigation of brittle failure in ductile, notch-sensitive thermoplastics. Polym. Eng. Sci..

[26] Fleischhauer R., Dal H., Kaliske M., Schneider K. (2012). A constitutive model for finite deformation of amorphous polymers. Int. J. Mech. Sci..

[27] Estevez R., Tijssens M.G.A., van der Giessen E. (2000). Modeling of the competition between shear yielding and crazing in glassy polymers. J. Mech. Phys. Solids.

[28] Fraser R.A.W., Ward I.M. (1977). The impact fracture behaviour of notched specimens of PC. J. Mater. Sci..

[29] Newmann L.V., Williams J.G. (1980). A comparative study of the tensile and charpy impact tests from a fracture mechanics viewpoint. Polym. Eng. Sci..

[30] Gearing B.P. (2002). Constitutive Equations and Failure Criteria for Amorphous Polymeric Solids by Submitted to the Department of Mechanical Engineering. https://dspace.mit.edu/handle/1721.1/17543.

[31] Torres J.P., Frontini P.M. (2016). Mechanics of PC in biaxial impact loading. Int. J. Solids Struct..

[32] Gearing B.P., Anand L. (2004). Notch-sensitive fracture of PC. Int. J. Solids Struct..

[33] Gearing B.P., Anand L. (2004). On modeling the deformation and fracture response of glassy polymers due to shear-yielding and crazing. Int. J. Solids Struct..

[34] Kattekola B., Ranjan A., Basu S. (2013). Three dimensional finite element investigations into the effects of thickness and notch radius on the fracture toughness of PC. Int. J. Fract..

[35] Dorogoy A., Rittel D., Brill A. (2011). Experimentation and modeling of inclined ballistic impact in thick PC plates. Int. J. Impact Eng..

[36] Husain A., Ansari R., Chan A.H. (2016). Experimental and numerical investigation of perforation of thin polycarbonate plate by projectiles of different nose shape. Lat. Am. J. Solids Struct..

[37] SIMULIA (2009). Abaqus User Manual. Ver 6.9. http://130.149.89.49:2080/v6.9ef/books/usi/default.htm.

[38] Pouzada A.S., Ferreira E.C., Pontes A.J. (2006). Friction properties of moulding thermoplastics. Polym. Test..

[39] Lee J.H., Xu G.H., Liang H. (2001). Experimental and numerical analysis of friction and wear behavior of PC. Wear.

[40] Richeton J., Ahzi S., Vecchio K.S., Jiang F.C., Adharapurapu R.R. (2006). Influence of temperature and strain rate on the mechanical behavior of three amorphous polymers: Characterization and modeling of the compressive yield stress. Int. J. Solids Struct..

[41] Blumenthal W.R. (2003). Influence of Temperature and Strain Rate on the Compressive Behavior of PMMA and PC Polymers. AIP Conf. Proc..

[42] Sabet S.A., Borst R. (2019). Structural softening, mesh dependence, and regularisation in non-associated plastic flow. Int. J. Numer. Anal. Methods Geomech..

[43] Kanani A.Y., Liu Y., Hughes D.J., Ye J., Hou X. (2020). Fracture mechanisms of hybrid adhesive bonded joints: Effects of the stiffness of constituents. Int. J. Adhes. Adhes..

[44] Campilho R.D.S.G., Fernandes T.A.B. (2015). Comparative evaluation of single-lap joints bonded with different adhesives by cohesive zone modelling. Procedia Eng..

